# Human Amniotic Membrane for Dural Repair and Duraplasty: A Systematic Review of Safety and Efficacy

**DOI:** 10.7759/cureus.51117

**Published:** 2023-12-26

**Authors:** Abdallah Abbas, Abdullah A Hamad, Osam O Ballut, Rawan M El-Gayar, Ahmed Negida, Ahmed M Raslan

**Affiliations:** 1 Neurology, Faculty of Medicine, Al-Azhar University, New Damietta, EGY; 2 Neurology, Medical Research Group of Egypt, Negida Research Academy, Arlington, USA; 3 Neurology, Faculty of Medicine, Menoufia University, Shibin El-Kom, EGY; 4 Neurology, Faculty of Medicine Kasr Al-Ainy, Cairo University, Cairo, EGY; 5 Neurology, Faculty of Medicine, Zagazig University, Zagazig, EGY; 6 Neurology, Virginia Commonwealth University, Richmond, USA; 7 Pharmacy and Biomedical Sciences, University of Portsmouth, Portsmouth, GBR; 8 Neurological Surgery, Oregon Health & Science University, Portland, USA

**Keywords:** human amniotic membrane, dural repair, dural substitutes, dura mater, duraplasty, amnion

## Abstract

The use of human amniotic membrane (HAM) has recently gained attention as a promising alternative option for duraplasty due to its superior tensile strength, elasticity, and anti-inflammatory and anti-fibrotic properties, offering greater durability and reliability compared to autologous grafts like the muscle fascia and pericranium. This systematic review aimed to evaluate the complications associated with duraplasty using HAM. We comprehensively searched the PubMed, Scopus, and Web of Science databases for studies on duraplasty with HAM. The eligibility criteria included studies on patients who underwent dural repair with duraplasty using HAM, with or without a control group. Duraplasty involves opening the dura mater, the protective covering of the brain and spinal cord, and using a graft to enlarge the space around the cerebellum. Dual repair, on the other hand, involves repairing the dura mater without opening it and then using a patch to enlarge the space around the cerebellum. Randomized controlled trials, observational studies, case series, and case reports were included, and quality assessment was conducted. Our search yielded 191 articles. Ten studies were included, with a total of 560 participants. The overall incidence of cerebrospinal fluid (CSF) leakage was three (0.63%) out of 478 in the HAM group and three (4.76%) out of 63 in the other methods group (pericranium, temporalis fascia, and biological dural substitutes). Regarding the incidence of postoperative complications, the overall incidence was eight (1.92%) out of 417 in the HAM group and two (8%) out of 25 in the other methods group. The overall incidence of meningitis was one (0.67%) out of 150 in the HAM group and three (10%) out of 30 in the other methods group. In conclusion, duraplasty using HAM may be a safe and effective alternative to traditional methods, with a low incidence of CSF leakage and postoperative complications.

## Introduction and background

Duraplasty is a surgical operation to reconstruct the open dura mater after it has been cut open during surgery [1]. It could be performed using autologous grafting (fascia lata, temporalis fascia, etc.), xenograft (bovine pericardium), or synthetic (collagen matrix, etc.) [1]. It is also utilized in a variety of clinical scenarios, including but not limited to watertight closure, expansile, or brain coverage/dural enforcement [2]. Due to the inherent shortcomings associated with each of these methods, the exploration of materials continues [2].

The human amniotic membrane (HAM) has been used to treat several medical conditions, including ocular surface disease, corneal ulcers, oropharyngeal disorders, and chronic wounds [3,4]. Its immunosuppressive and anti-inflammatory properties provide it with therapeutic potential [5]. It has also been proposed as a matrix for the tissue engineering of epithelial tissues and a source of stem cells for regenerative medicine, among other applications [5]. There are potential additional benefits to the use of HAM, including the following: 1) there is improved resistance to adherent cerebrospinal fluid (CSF) leakage, 2) there is reduced risk of obliteration of the subarachnoid space, 3) its availability in multi-layered sheets allows for better coverage after dural defects, 4) the preservation of pain pathways from trauma passing through the tissue remains intact due to its minimal cellularity, and 5) there is a lower risk of autoimmune response since HAM does not contain cells; foreign body reactions are also reduced [6-8].

Regarding neurosurgical applications, HAM has been used as a dural substitute in various neurosurgical procedures (skull base surgery, craniotomies, trauma surgery, spine surgery, and duraplasty) [6,9,10] due to its biocompatibility, resistance to infection, and lack of inflammation [9]. HAM has been shown to promote faster healing and reduced adhesions, as well as a decrease in the rate of post-surgical complications when compared with the muscle fascia or pericranium [9].

In a clinical trial, HAM was used as a substitute for dural repair after skull base surgery, and it was found to be useful in preventing CSF leakage and pseudomeningocele [9]. Another study by Marton et al. [11] found that HAM can be safely utilized as an adjunct during dural closures for craniotomies and enhances the dura's ability to create a watertight seal.

The use of HAM in duraplasty is still a subject of research and controversy among neurosurgeons. Some studies showed that it was more effective than the other well-known materials [7]. On the other hand, other studies showed the opposite [6]. So, we conducted this systematic review to provide an overview of the studies published on this topic and a quality assessment of these studies and also to compare HAM grafts to other known dural graft materials to demonstrate the clinical efficacy of these grafts in dural repair in neurosurgery.

## Review

Methods

We followed the Preferred Reporting Items for Systematic Reviews and Meta-Analyses (PRISMA) guidelines in reporting this systematic review. This review was prospectively registered in Prospero (CRD42023395320).

Selection Criteria

Our study comprised cases where patients underwent duraplasty with HAM and intracranial surgery. The comparator or other methods were pericranium, temporalis fascia, or biological dural substitutes or even no comparator. We included randomized controlled trials, case-control studies, cohort studies, case reports, and case series while excluding reviews and meta-analysis studies.

Literature Search

We searched databases (PubMed, Scopus, and Web of Science) from inception until October 2023 for articles related to our topic using the following search strategy: ((duraplasty) OR (dura) OR (dura mater) OR (dural)) AND ((amnion) OR (amniotic)). We didn't use any filters during the search process.

Screening and Data Extraction

Four authors conducted an independent title and abstract screening followed by a full-text screening of the study, and any inconsistencies were referred to a fifth author. The data extraction process was conducted by four authors independently, and a fifth author reviewed it. In addition to extracting the baseline characteristics (age, sex, and neurosurgical procedures) of the included studies, we collected all complications related to the utilization of HAM for duraplasty, which included postoperative complications, meningitis, wound infections, and CSF leakage.

Quality Assessment

Two independent authors evaluated the quality of the studies included in our analysis, with any discrepancies being referred to a third author. The Joanna Briggs Institute (JBI) [12] assessed case series; the Newcastle-Ottawa scale (NOS) [13] evaluated observational cohort studies; and the revised Cochrane risk-of-bias tool (RoB 2) [14] assessed randomized controlled trials. JBI is composed of 10 questions, with a response of yes, no, not clear, or not applicable and an overall score of 10. The NOS tool evaluated each case-control study for nine items, which are divided into three groups: the selection of the study participants, the comparability of the groups, and the ascertainment of either the exposure or outcome of interest, with a yes or no response to each item. Studies with scores of 7-9 are deemed high quality, 4-6 are of moderate quality, and 1-3 are of low quality. The RoB 2 [14] is separated into five domains, each with a set of questions. These questions have a response of yes, no, possibly yes, possibly no, and no information. The results are combined through a diagram to give one of three levels of bias: low risk, some concern, or high risk of bias. If the five domains have a low risk of bias, then the study has a low risk of bias. If at least one domain has some concerns, then the study is reported to have some concerns about bias. If at least one domain has a high risk of bias or multiple domains have some concerns, then the study is reported to have a high risk of bias.

Results

Search and Selection Method

**Figure 1:**
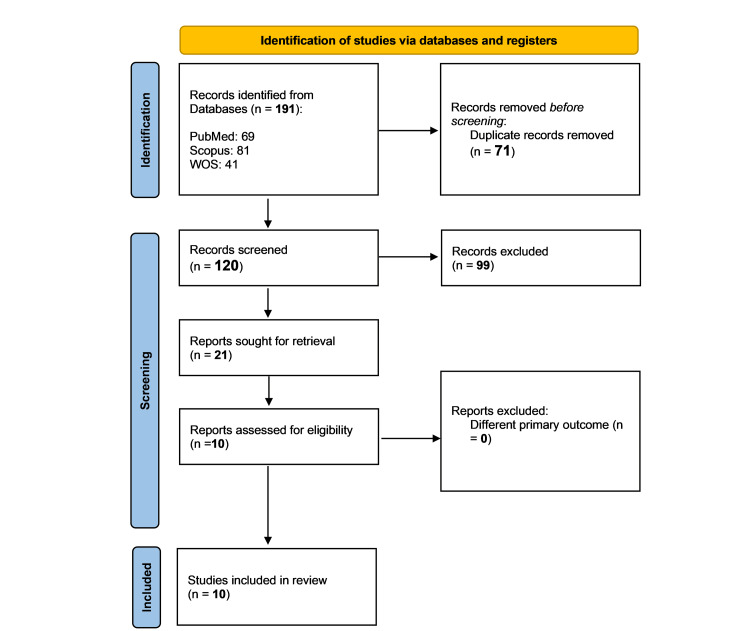
The PRISMA flow diagram of the search and screening process results PRISMA: Preferred Reporting Items for Systematic Reviews and Meta-Analyses; n: number of studies; WOS: Web Of Science

By searching the databases we utilized, we were able to find 191 research publications. After removing duplicates, we had 120 articles that needed to be evaluated. After assessing their titles, abstracts, and complete texts, we narrowed down to 10 studies [6-11,15-18] that fit our criteria and were eligible for systematic review, as shown in Figure 1.

Quality Assessment

The overall results of the quality assessment of the 10 studies revealed that there were four studies [9-10,15-16] with high quality, five studies [6,8,11,17-18] with good quality, and one study [7] with a high risk of bias. Four studies [9-10,15-16] were assessed by the JBI tool. Three studies of them [10,15-16] received a score of 8 out of 10, and one study [9] got 9 out of 10, as shown in Table 1.

**Table 1:** The quality assessment of the case series studies according to JBI JBI: Joanna Briggs Institute

	Al Fauzi et al. [15]	Marton et al. [16]	Walker et al. [10]	Tomita et al. [9]
1. Were there clear criteria for inclusion in the case series?	Yes	Not clear	Not clear	Not clear
2. Was the condition measured in a standard, reliable way for all participants included in the case series?	Yes	Yes	Yes	Yes
3. Were valid methods used for the identification of the condition for all participants included in the case series?	Yes	Yes	Yes	Yes
4. Did the case series have consecutive inclusion of participants?	Yes	Yes	Yes	Yes
5. Did the case series have a complete inclusion of participants?	Yes	Yes	Yes	Yes
6. Was there clear reporting of the demographics of the participants in the study?	Not clear	Yes	Yes	Yes
7. Was there clear reporting of clinical information of the participants?	Not clear	Yes	Yes	Yes
8. Were the outcomes or follow-up results of cases clearly reported?	Yes	Yes	Yes	Yes
9. Was there clear reporting of the presenting site(s)/clinic(s) demographic information?	Yes	Not clear	Not clear	Yes
10. Was statistical analysis appropriate?	Yes	Yes	Yes	Yes
Overall	8 out of 10	8 out of 10	8 out of 10	9 out of 10

Using the NOS tool, five studies [6,8,11,17-18] were assessed as having good quality, as illustrated in Table 2.

**Table 2:** The quality assessment of the observational cohort studies according to NOS NOS: Newcastle-Ottawa scale; NA: not applicable

Study	Selection				Comparability	Outcome			Quality
	Representativeness of the exposed cohort	Selection of the non-exposed cohort	Ascertainment of exposure	Outcome of interest was not present at the start of study		Assessment of outcome	Was follow-up long enough for outcomes to occur	Adequacy of follow-up of cohorts	
Marton et al. [11]	0	⭐	⭐	⭐	⭐	⭐	0	⭐	Good quality
Tahami et al. [6]	0	⭐	⭐	⭐	⭐	⭐	0	⭐	Good quality
Eichberg et al. [17]	⭐	NA	⭐	⭐	NA	⭐	⭐	⭐	Good quality
Eichberg et al. [8]	⭐	NA	⭐	⭐	NA	⭐	⭐	⭐	Good quality
Eichberg et al. [18]	⭐	NA	⭐	⭐	NA	⭐	⭐	⭐	Good quality

In RoB 2 of Turchan et al. [7], three domains were high, and two were high, resulting in an overall high score, as shown in Table 3.

**Table 3:** The quality assessment of the RCT studies according to RoB 2 RCT: randomized controlled study; RoB 2: revised Cochrane risk-of-bias tool; D1: randomization processes; D2: deviating from the intended intervention; D3: missing outcome data; D4: measurement of the outcome; D5: selection of the reported results

	D1	D2	D3	D4	D5	Overall
Turchan et al. [7]	High	Low	Low	Low	High	High

Baseline Characteristics

Ten studies [6-11,15-18] with a total of 560 participants were included. There were various neurosurgical procedures, as shown in Table 4.

**Table 4:** Baseline and clinical characteristics of included studies n: number of patients; HAM: human amniotic membrane; SD: standard deviation; RCT: randomized controlled trial

Authors	Country	Study design	Control	Patients in the control group (controls) (n)	Patients with HAM dural repair (cases) (n)	Sex (males) n (%), cases, controls	Age: mean (SD) or mean (range) cases, controls	Neurosurgical procedure
Tahami et al. [6]	Iran	Prospective	Pericranium	30	30	Cases: 17 (56.7%); controls: 14 (46.7%)	Cases: 43.9 (19); controls: 39.1 (15)	Craniotomy: 30
Turchan et al. [7]	Indonesia	RCT	Temporalis fascia	8	8	Cases: 6 (75%); controls: 6 (75%)	Cases: 33 (9.9); controls: 35 (15.9)	Decompressive craniectomy followed by cranioplasty: 8
Walker et al. [10]	USA	Case series	-	-	14	Cases: 3 (21.42%)	Cases: 47.85 (26-73)	Myelomeningocele: 3; craniotomy for Chiari decompression: 4; spontaneous ventral tethering at T5: 1; spinal trauma at T10-11: 1; cervical meningioma status postresection (×2) and radiation therapy with tethering at C6-T1: 1; conus medullaris lipoma resection with tethering at T12: 1; conus medullaris arteriovenous malformation resection with tethering at T12: 1; cervical intramedullary cavernous malformation resection with tethering at C6-7: 1; thoracic intramedullary lipoma resection (×2) with tethering at T7-10: 1
Al Fauzi et al. [15]	Indonesia	Case series	-	-	8	-	-	Decompressive craniectomy followed by cranioplasty: 8
Eichberg et al. [17]	USA	Retrospective	-	-	122	Cases: 45 (36.9%)	Cases: 58 (86.25)	Initial supratentorial craniotomy: 102; initial infratentorial craniotomy: 18
Eichberg et al. [8]	USA	Retrospective	-	-	155	Cases: 62 (39%)	Cases: 57.2 (18)	Craniotomy: 122; transsphenoidal: 32; combined craniotomy and transnasal endoscopic: 1
Eichberg et al. [18]	USA	Retrospective	-	-	120	Cases: 54 (45%)	Cases: 53.5 (17.25)	Transsphenoidal endoscopic endonasal surgery: 120
Marton et al. [16]	Italy	Case series	-	-	5	Cases: 2 (40%)	-	Spinal dysraphism repair: 5
Marton et al. [11]	Italy	Prospective	Biological dural substitutes	25	25	Cases: 15 (60%); controls: 14 (46%)	Cases: 48.33 (33.01); controls: 41.33 (30.65)	Decompressive craniectomy followed by cranioplasty: 25
Tomita et al. [9]	Japan	Case series	-	-	10	Cases: 5 (5%)	Cases: 54.9 (5-71)	Skull base surgery: 10

Outcomes Measured in the Different Procedures Across the Studies

The HAM in craniotomy procedures: Three studies analyzed the effect of HAM on patients who underwent craniotomy [6,8,17] with a total of 273 patients. Tahami et al. [6] found that there was no significant difference in the occurrence of CSF leakage or pseudomeningocele between the group that underwent duraplasty using the amniotic membrane and the group that underwent duraplasty using pericranium as a dural graft. However, there was a significantly higher prevalence of underlying disease-induced hydrocephalus in patients who underwent posterior fossa craniotomy compared to supratentorial craniotomy (p<0.00001). Another pilot study by Eichberg et al. [17] revealed that the use of dehydrated amniotic membrane (DAM) allograft for dural repair in craniotomies is safe and effective, with no complications related to the use of DAM allograft reported in the study. The authors suggest that the use of DAM allograft may be a viable alternative to other materials commonly used in dural repair, such as synthetic dural substitutes or autologous tissue. However, the study was not randomized and lacked a control group, so the findings could be biased due to confounding variables. The interpretation of the data is also complicated by the fact that the patients in the study received a sheet of bovine collagen dural substitute layered on top of the DAM; thus, the outcomes may be due to both materials. Future studies with patients randomized into groups with other dural closure techniques are required to validate this study. Another study by Eichberg et al. [8] found the following: First, the use of DAM did not contribute to an increased risk of CSF leak in surgeries that are considered high risk for CSF leak, including the posterior fossa, sellar region, and anterior skull base. The study's series contained 52 surgeries in these locations, and none of them were complicated by a CSF leak. This preliminary evidence suggests that DAM does not increase the risk of CSF leaks. Second, the use of DAM was associated with a low rate of infection, with only one patient developing a surgical site infection out of the 155 patients included in the study.

The HAM in decompressive craniectomy and cranioplasty: Three studies examined the HAM in decompressive craniectomy followed by cranioplasty among 41 patients [7,11,15]. Turchan et al. [7] suggested that duraplasty healing using an amniotic membrane graft was as effective and safe as that using a fascial graft. The use of amniotic membrane graft was able to provide watertight dural closure and adequate fibrocyte infiltration for edge healing of the dura mater defect. The study also found that there were no significant differences in the incidence of complications or side effects between the two techniques. Therefore, the researchers concluded that amniotic membrane grafts had a potential advantage as a dural substitute and could be considered as an alternative to temporal muscle fascia grafts in certain cases. A study conducted by Marton et al. [11] in 2021 suggested that the homologous amniotic membrane can be a safe and effective substitute for the dura mater in decompressive craniectomies. The study found that the use of the amniotic membrane reduced the risk of adhesions to the brain parenchyma while avoiding CSF leakage and infections. Additionally, the study found that the amniotic membrane was able to integrate with the native dura during cranioplasties. Al Fauzi et al. [15] found that the amniotic membrane graft was able to provide a watertight effect while testing with 0.9% NaCl injections in patients who underwent decompressive craniectomy followed by cranioplasty. The amniotic membrane also showed the capability of stimulating adequate fibrocyte infiltration based on the histological review, thus supporting edge healing of the dura mater.

The use of HAM in spinal procedures: Two studies investigated the use of HAM in spinal procedures with a total of 15 patients [10,16]. A case series by Walker et al. [10] in 2018 examined the HAM in different spinal procedures among 10 patients (myelomeningocele: 3; spontaneous ventral tethering at T5: 1; spinal trauma at T10-11: 1; cervical meningioma status postresection (×2) and radiation therapy with tethering at C6-T1: 1; conus medullaris lipoma resection with tethering at T12: 1; conus medullaris arteriovenous malformation resection with tethering at T12: 1; cervical intramedullary cavernous malformation resection with tethering at C6-7: 1; thoracic intramedullary lipoma resection (×2) with tethering at T7-10: 1) which suggested that the use of HAM grafts is a safe and effective technique for preventing intradural spinal cord adhesions. The authors found that none of the patients who received the grafts experienced any complications related to the use of HAM. Additionally, they observed a significant reduction in tethering caused by adhesions in all patients who received the grafts. The authors concluded that HAM grafts represent a novel approach to addressing this difficult clinical problem and further studies are needed to evaluate their long-term efficacy. Another case series by Marton et al. [16] examined five patients who underwent spinal dysraphism repair and revealed that no adverse events occurred and the surgical wounds healed without complications. MRI scans taken at three and six months after the surgery showed a satisfying de-tethering of the spinal cord with no obvious evidence of new adherence formation. HAM proved its efficacy in restoring dural sac integrity without complications.

The HAM in transsphenoidal procedures: Two studies with a total of 152 patients retrospectively analyzed the patients who underwent transsphenoidal surgery using HAM [8,18]. The two studies conducted by Eichberg et al. [8] found that the use of DAM allograft can be safely utilized as an adjunct during sellar closures for transsphenoidal endoscopic endonasal surgery (TEES) for pituitary adenoma resection with very low rates of CSF leak and meningitis. In a cohort by Eichberg et al. [18] of 120 patients with a 49.2% intraoperative CSF leak rate, the postoperative CSF leak rate was only 1.7% for patients who received DAM and 0.9% for TEES-naïve patients. The study found an adequate safety profile with no adverse reactions directly related to the DAM product.

The HAM in skull base surgery: In a case series by Tomita et al. [9], involving 10 patients who underwent skull base surgery, the authors suggested that the DAM can serve as an alternative to autologous tissue for dural repair, demonstrating its effectiveness in preventing CSF leakage after skull base surgery. Additionally, the study reported histological changes in the implanted dried amniotic membrane and surrounding tissue over time.

Discussion

The HAM has been used in various medical procedures due to its unique properties that promote healing and tissue regeneration. Compared to other methods, the use of HAM has shown several benefits, including reduced inflammation, improved wound healing, and increased tissue regeneration [19,20]. In neurosurgery, HAM has been used to repair dura mater defects, promote spinal cord healing, and treat peripheral nerve injuries [10,11]. The use of HAM in these procedures has shown promising results, with studies reporting improved outcomes compared to traditional methods [10,11]. This report has focused on the use of HAM for dural repair and/or duraplasty in particular.

HAM has several advantages over autologous grafts, such as the muscle fascia and pericranium in duraplasty. HAM has higher tensile strength and elasticity, which allows for greater durability and reliability compared to the muscle fascia and pericranium [7,21]. Additionally, HAM has anti-inflammatory and anti-fibrotic properties that can reduce inflammation and scarring at the site of the repair, leading to faster healing and a lower risk of complications [22]. When it comes to xenograft and synthetic dural substitutes, HAM has several advantages over both. Firstly, the HAM is highly biocompatible and has low immunogenicity [7,23,24]. Secondly, it has anti-inflammatory properties and can reduce scarring [25,26]. Thirdly, it can act as a natural scaffold for tissue regeneration and contains several types of stem cells and potent growth factors [7,23,27,28]. Fourthly, it has been shown to have comparable outcomes to commercially available dural substitutes [28,29]. In contrast, autologous grafts, such as the muscle fascia and pericranium, can cause damage to donor sites and have limitations in size and shape [30]. Xenografts can be associated with adverse effects such as graft dissolution, encapsulation, foreign body reactions, scarring, and infection [7,28,30]. Synthetic dural substitutes, such as DuraGen (Integra LifeSciences, Princeton, New Jersey, United States), can have drawbacks such as graft degradation, weak tissue over the graft, and higher antigenicity [7,28,30]. Therefore, the HAM is a promising alternative to autologous grafts, xenografts, and synthetic dural substitutes in duraplasty due to its superior biological properties and lower risk of complications.

Our study investigated the efficacy of using HAM as a dural substitute in duraplasty procedures. Our results suggest that using HAM as a dural substitute may reduce the incidence of CSF leakage in comparison to other methods (pericranium, temporalis fascia, and biological dural substitutes), as shown in Table 5. Additionally, the incidence of postoperative complications was low in the HAM group across multiple studies, as illustrated in Table 5. Given that postoperative complications and CSF leakage can complicate duraplasty procedures, these findings have significant implications for improving patient outcomes. Furthermore, our study adds to the growing body of literature on the use of HAM in neurosurgery and may inform future clinical practice by providing evidence for the use of HAM in duraplasty procedures.

**Table 5:** The incidence of safety outcomes in the HAM group in comparison with other methods CSF: cerebrospinal fluid; HAM: human amniotic membrane; n: number of patients

Safety outcomes	HAM group (n%)	Other methods (pericranium, temporalis fascia, and biological dural substitutes) (n)
CSF leakage	3 (0.63%) out of 478 (100%)	3 (4.76%) out of 63 (100%)
Postoperative complications	8 (1.92%) out of 417 (100%)	2 (8%) out of 25 (100%)
Meningitis	1 (0.67%) out of 150 (100%)	3 (10%) out of 30 (100%)
Wound infections	2 (0.72%) out of 277 (100%)	-
Major inflammatory response	0 (0%) out of 120 (100%)	-

These findings are consistent with several studies that have suggested the use of HAM in neurosurgical procedures. Shah et al. [28] led a review on how well the HAM works as a substitute for dura. However, their study has some limitations that need to be addressed. For example, they didn't include two important studies in their analysis, which reduced the number of studies they looked at to only eight. This happened because they didn't do a thorough search. Also, they didn't have a plan for their study, or they did not register the protocol for this study, which makes it hard to understand how they did it. This study [28] found that HAM was associated with a low rate of complications, such as CSF leaks; Turchan et al. found that the use of the HAM graft was able to provide watertight dural closure and adequate fibrocyte infiltration for edge healing of the dura mater defect [7]. However, some studies have reported no significant difference in the incidence of complications between HAM and other methods. Tahami et al. revealed that there was no significant difference in the occurrence of CSF leakage or pseudomeningocele between the group that underwent duraplasty using HAM and the group that underwent duraplasty using pericranium as a dural graft [6]. On the other hand, patients who underwent a posterior fossa craniotomy exhibited a significantly higher prevalence of underlying disease-induced hydrocephalus than those who underwent a supratentorial craniotomy (p<0.00001).

The strength of our study design lies in the fact that we followed PRISMA guidelines and screened the databases for relevant articles. We included various study designs, including randomized controlled trials, case-control studies, cohort studies, and case series, with a total of 560 participants. We also assessed the quality of the included studies using validated tools. However, our study has some limitations that need to be considered. Firstly, the included studies were heterogeneous in terms of the patient population, surgical procedures, and the use of HAM, which could have introduced bias into our analysis. Secondly, most of the included studies were single-arm studies, which limits the strength of our conclusions. Thirdly, there was a lack of consistency in the definitions and reporting of outcomes across the included studies, which may have affected our ability to make meaningful comparisons. Finally, there is a lack of long-term follow-up on the effect of HAM in duraplasty. While the review highlights the short-term benefits of using HAM, such as the reduced risk of CSF leakage and postoperative complications, there is a need for further research to evaluate its long-term effects. Specifically, studies should focus on evaluating the long-term efficacy and safety of HAM, including its potential to cause adverse reactions, infections, and rejection.

## Conclusions

Our study suggests that the use of HAM in duraplasty is a safe and effective alternative to other recognized dural graft materials. The use of HAM was associated with a lower risk of CSF leakage, wound infection, major inflammatory response, and meningitis. Further studies are needed to confirm our findings and address the heterogeneity among the studies.
